# Blood Donations and Transfusions during the COVID-19 Pandemic in Spain: Impact According to Autonomous Communities and Hospitals

**DOI:** 10.3390/ijerph18073480

**Published:** 2021-03-27

**Authors:** José Antonio García-Erce, Íñigo Romón-Alonso, Carlos Jericó, José María Domingo-Morera, José Luis Arroyo-Rodríguez, Carlos Sola-Lapeña, José Luis Bueno-Cabrera, Raúl Juárez-Vela, Saioa Zalba-Marcos, Ane Abad-Motos, Vicente Gea-Caballero, Iván Santolalla-Arnedo, Manuel Quintana-Díaz

**Affiliations:** 1 Banco de Sangre y Tejidos de Navarra, Servicio Navarro Salud, Grupo Español de Rehabilitación Multimodal (GERM), IACS, Zaragoza, Grupo IdiPaz. Madrid, 31008 Pamplona, Spain; ja.garcia.erce@navarra.es; 2 Servicio Hematología y Hemoterapia, Universitario Marqués de Valdecilla, 39003 Santander, Spain; iromon@humv.es; 3 Hospital Sant Joan Despí de Barcelona Servicio de Medicina Interna, 08970 Barcelona, Spain; cjericoalba@gmail.com; 4 Banco de Sangre y Tejidos de Aragón, 5001 Zaragoza, Spain; jmdomingom@aragon.es; 5 Banco de Sangre y Tejidos de Cantabria, 39008 Santander, Spain; director@bscan.org; 6 Centro y Servicio de Transfusión de la Rioja, 26006 Logroño, Spain; csola@riojasalud.es; 7 Servicio Hematología-Hemoterapia, Hospital Universitario Puerta de Hierro, 28222 Majadahonda-Madrid, Spain; jolubuca@telefonica.net; 8 Departamento de Enfermería, Universidad de la Rioja, Centro de Investigación Biomédica de La Rioja CIBIR-GISOSS, 26004 Logroño, Spain; 9 Servicio Hematología-Hemoterapia, Complejo Hospitalario de Navarra, 31008 Pamplona, Spain; saoia.zalba.marcos@navarra.es; 10 Servicio Anestesia y Reanimación, Hospital Infanta Leonor, 28031 Madrid, Spain; aneabad@hotmail.com; 11 Health Research Institut La Fe, Adscript Center Universidad de Valencia, Nursing School La Fe, 46026 Valencia, Spain; gea_vic@gva.es; 12 Servicio de Medicina Intensiva, Hospital Universitario La Paz, Grupo IdiPaz. Madrid, 28046 Madrid, Spain; mquintanadiaz@gmail.com

**Keywords:** COVID-19, transfusion, pandemic

## Abstract

Worldwide, the COVID-19 pandemic has caused a decline in blood donations, between 30% and 70% in some of the most affected countries. In Spain, during the initial eight weeks after the State of Emergency was decreed on 14 March 2020, in the weekly reports of the Health Ministry, an average decrease of 20% was observed between 11 and week 25 compared with the 2018 donation. We aimed to investigate the impact of the COVID-19 pandemic on blood donations and blood distribution in four autonomous communities, and to explore the evolution of the consumption of blood components (BCs) in ten hospitals of six autonomous communities. We performed a prospective study of grouped cohorts on the donation and distribution of blood in four regional transfusion centers in four autonomous communities in Spain, and a retrospective study of the consumption of blood components in ten hospitals in six autonomous communities. Regarding donations, there was no significant decrease in donations, with differences between autonomous communities, which started between 1 and 15 March 2020 (−11%). The increase in donations in phase II (from 26 May 2020) stands out. Regarding consumption, there was a significant reduction in the consumption of packed red blood cells (RBCs) (24.5%), plasma (45.3%), and platelets (25.3%) in the central period (16 March–10 May). The reduction in the consumption of RBCs was significant in the period from 1–15 March. Conclusions: The COVID-19 pandemic has affected the donation and consumption of BCs.

## 1. Introduction

During the previous pandemics of the current century, many countries reported a shortage of blood components (BCs) due to a decline in donations [1]. After the declaration of the COVID-19 pandemic worldwide, some authors warned about the potential risks of transfusion activity [2] including shortages (decreased donations due to illness in the population, lack of health personnel at donation centers, lack of supplies, etc.); the risk of the transmission of infection by blood transfusion (in turn facilitated by the lack of sensitive and specific rapid tests for the screening of donors or donations, with the need for the implementation of inactivation procedures or the improvement of existing ones); and a potential increase in consumption due to patient need or due to a change in transfusion practice. Thus, in the first weeks of the pandemic, donations declined between 20% to 67% in the provinces of China [3,4,5], India [1,6], Saudi Arabia [7], Iran [8], The Netherlands, southern Italy [9] and the Washington State, USA [10].

In Spain, the Scientific Committee on Transfusion Safety of the Ministry of Health urged the regional transfusion centers (RTCs) to promote measures to ensure stocks in the event of a probable drop in blood donations [11]. In addition, the committee encouraged weekly communication about the situation of BC donations and deposits. The RTCs established contingency plans, and some hospitals instituted measures to reduce and adapt the consumption of BCs.

The state of health alert and the state of national alarm with confinement in Spain (14 March 2020) threatened transfusion activity. On the one hand, the drop in donations and the adjustment of calls for donors guaranteeing security posed a new challenge for the RTCs. On the other hand, the suspension of most delayed programmed surgical activity during the first wave in Spain [12], a decrease in admissions due to multiple injuries, and a reduction in all organ transplantation activity [13] augured a foreseeable decrease in the consumption of BCs [9,10]. However, there are no published studies on the real impact of a pandemic on donation and consumption in various regions in a time lapse longer than several weeks or a month, or on the different phases of the pandemic beyond experiences isolated to a specific region, or in a major part of or an entire country.

For this reason, we propose this study whose main objective is to describe how the COVID-19 pandemic has influenced the number of donations, distribution, and blood transfusions in different RTCs and hospitals of different autonomous communities in the different phases of the state of alarm decreed by the pandemic (including a period of two months before it), and to compare the results obtained with the transfusion activity of the two years before the pandemic in Spain. For this, we intend to specifically evaluate the rate of donation, distribution, and transfusion of different BCs (packed red blood cells, platelets, and fresh frozen plasma) in the different autonomous communities and hospitals according to the grade of interterritorial affectation by the pandemic.

## 2. Materials and Methods

### 2.1. Study Design

A prospective study of pooled cohorts was conducted on the donation and distribution of blood in four regional transfusion centers of four autonomous communities and a retrospective observational study was conducted on the consumption of BCs in ten hospitals of six autonomous communities, comparing periods of different phases of the state of alarm due to the COVID-19 pandemic and the previous months, with the average consumption in the same period of the 2018–2019 biennium.

### 2.2. Participants/Scope of the Study

RTCs and transfusion services and/or transfusion committees of different autonomous communities were invited to participate in the study. Finally, four regional RTCs and 10 hospitals from 6 autonomous communities were selected (Aragón, Cantabria, La Rioja, Navarra, Madrid, and Catalonia). An autonomous community is a region of Spain endowed with autonomy, with its own institutions and representatives. These regions were selected, which confirmed their ability to obtain data in the requested study period, as a criterion for unique inclusion.

The participating hospitals are level III–IV, which means that they have an intensive care, with cardiac surgery, hematopoietic transplantation, organ and tissue transplantation systems. Most hospitals have a reference population of between 350,000 and 500,000 patients. Most of them are referral hospitals in the autonomous community.

### 2.3. Period Studied

The period of greatest hospital involvement by the COVID-19 pandemic in Spain was analyzed, taking as a reference the following different phases of health alert: Phase 0 (from 16 March to 10 May 2020), Phase 1 (11–24 May 2020), and Phase 2 (25 May to 8 June 2020). The results obtained were compared with those of the same periods for the years 2019 and 2018.

### 2.4. Study Variables

Data on donation and distribution were obtained from participating RCTs (Aragon, Cantabria, La Rioja, and Navarra) and from in-hospital donations from hospitals in Madrid and Catalonia. The number of patients and units of each blood component (red blood cells (RBCs), platelets, and fresh frozen plasma) transfused in each center during each of the study periods defined within the year 2020 (COVID-19 pandemic period) were collected as transfusion variables, and the same variables in the same periods for the years 2019 and 2018. The appearance of shortages and hemovigilance were also studied to monitor a possible hematic transmission of SARS-CoV-2.

As secondary variables, information was obtained from the participating hospitals (level of care and number of beds) and their activity (number of hospital admissions, surgical interventions with admission, and attendance at hospital emergencies).

### 2.5. Data Collection

The population data were obtained from the National Institute of Statistics (www.ine.es) (Visited February 2021).

Historical data on donation and transfusion nationally and by autonomous communities were obtained from the last available official Annual Activity Report from 2018 by the Ministry of Health [14].

To calculate the impact on transfusion, the disaggregated data on the hospital and transfusion activity of the hospitals where the participating RCTC and hospitals operate were extracted from the hospital records.

Data on national blood donations were obtained from the weekly monitoring reports of the Scientific Committee on Transfusion Safety, collected from Week 11 (9 to 15 March) through to Week 26 (22 to 28 June 2020). The data in absolute numbers of the amount of blood components were collected by period and centers.

The participating researchers collected the data from their CRTs or hospitals, while the coordinating researchers independently verified the consistency of the data, their input to the global database, and performed the statistical analysis.

A unified Excel template (Microsoft Suite, Microsoft, Redmont, Washington, DC, USA) was used for data collection.

### 2.6. Statistical Analysis

After data collection, the data were verified for integrity, the database was cleaned, and the analysis was performed using IBM SPSS v25.0 (Yuba City, CA, USA).

The impact of the pandemic was calculated using the average donations in 2018 as a reference. These reports provided information on the number of autonomous communities with a relative increase or decrease in the consumption of various BCs.

For each center and period analyzed, the percentage change in the value of the year 2020 of those of the different variables analyzed concerning the mean of the years 2018 and 2019 was calculated.

The statistical significance of the mean percentage change by centers was contrasted using a *t*-test for a sample, assuming a significance level of α = 0.05. To analyze the evolution of the periods of the year 2020 for each center, the values of the first period were taken as a reference (value 100), showing the evolution concerning the initial reference and adjusting for the duration of the periods, and the seasonality of Holy Week and the first week of January.

## 3. Results

### 3.1. Activity in Spain

According to the compilation of reports from the Ministry of Health (not published), during the study period, 196,245 donations were produced, which was 50,795 (−20.56%) less than the expected 247,040 according to the weekly reference average of the Ministry. The decrease was not homogeneous (Table 1), with an estimated decrease in donations of −25.88% in Phase 0, and an estimated increase in donations of 6.06% and 3.10% in Phases 1 and 2, respectively.

### 3.2. Donations

The four participating RTCs serve a population of 2,885,215 inhabitants estimated by the National Statistical Institute on 1 January 2020, i.e., 6.12% of the Spanish population. In the four regions, there was a homogeneous decline in blood donations as compared with the 2018–2019 period (Table 2). The four RTCs presented a non-significant decrease in Phase 0, with an average decrease of −20.1%, which was greater in those with the greatest impact of the pandemic.

**Figure 1:**
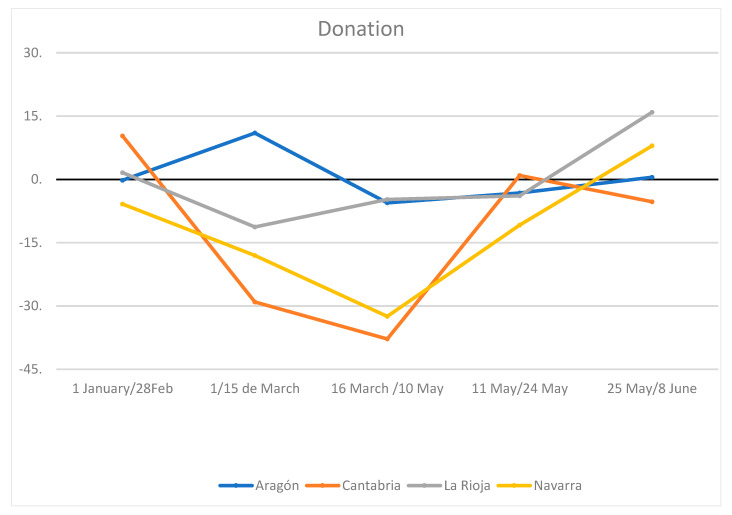
Evolution of donations. Percentage increase or decrease compared to the same time period of the previous biennium.

Three of the centers presented a decrease in donations in the fortnight before confinement (average −11.8%). Two of the centers showed an increase in donations in Phase 2 (average 4.8%). These changes, whether analyzed by taking the phases of the pandemic separately or by grouping them, did not show statistically significant differences (DES) due to differences between communities. Figure 1 reflects the impact of the drop and rebound of the donations for each CRT concerning the average of the same period in the previous 2 years.

In several of the hospital centers, donations were suspended due to reorganization, and redirected to their RTCs, which we have omitted from the analysis.

In Cantabria and Navarra, two of the autonomous communities with the highest rate of donation, but also of consumption, being two of the regions with the highest income per capita in Spain, the decrease was most significant, not justified by the impact on the pandemic, because in Cantabria the impact was much lower, followed by Aragon, while La Rioja and Navarra have been two of the regions with the highest infection and mortality rate from COVID. The data invite us to confirm that part of the explanation is the great variability of the consumption of blood components by the autonomous communities.

### 3.3. Distribution

The distribution of the BCs of the participating RTCs was not homogeneous among them and did not accord to the phases of the pandemic. A statistically significant decrease in the delivery of RBCs was observed for all centers in Phase 0 (−24.4%, *p* = 0.015) and in the previous fortnight (−18.6%, *p* = 0.019) (Appendix A, Table A1). In contrast, in Phases 1 and 2, there were differences between centers.

A statistically significant homogeneous decrease was not found in the supply of platelets, due to the differences between the RTCs; one center during the phases decreased by 19.1%, while in another there was an increase of 4.7% (Appendix A, Table A2).

Fresh frozen plasma was the component with the greatest drop in demand, over 40%, although a statistical significance was also not found. It should be noted that during the first two months of the year a decrease of −27.9% had been observed (95% CI −56.2, 2.4; *p* = 0.057). During the isolation period, the decline ranged from 51% to 85% (Appendix A, Table A3).

### 3.4. Consumption of Blood Components

During Phase 0, a global reduction with statistical significance in the transfusion of BC (−24.5%, 95% CI −30 to −19.1, *p* < 0.001), of platelets (−25.3%, 95% CI −36.5 to −14.1, *p* < 0.001) and fresh frozen plasma (−45.3%, 95% CI −76.7 to −14.6, *p* = 0.009) (Table 3).

In the fortnight prior to confinement (1–15 March), a drop in the transfusion of BCs with statistical significance was observed (−17.6%, 95% CI −28.2, to −7.1; *p* = 0.004), without significant changes in platelet and fresh frozen plasma consumption.

However, regarding platelet transfusions, it is in the last phase of the alarm state when a statistical significance is observed (−22.9%, 95% CI −36.4 to −9.4, *p* = 0.005). Table 4 shows the evolution of the BCs of the participating hospitals.

**Figure 2:**
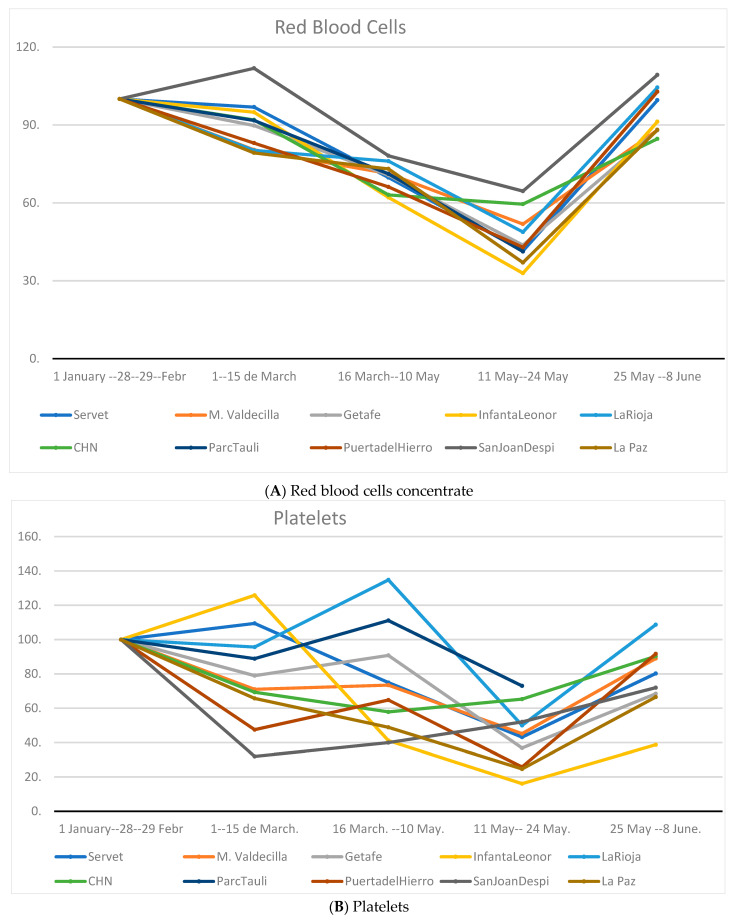

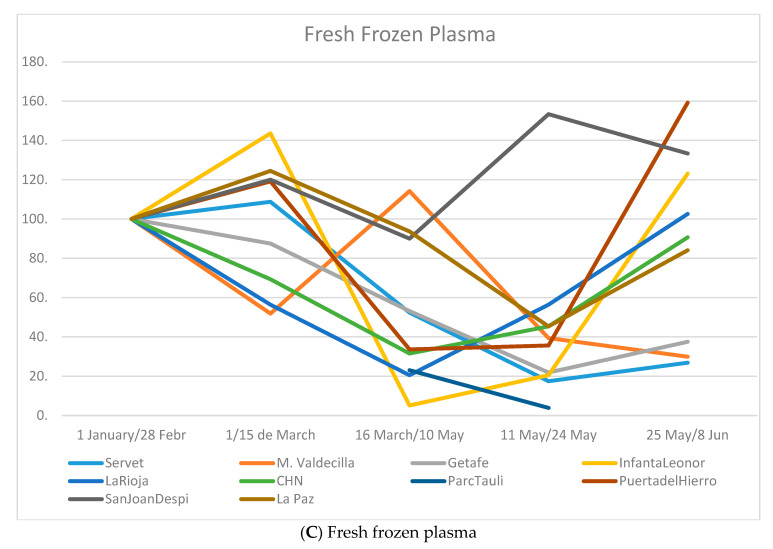
Evolution of blood component transfusion in adjusted hospital centers. CHN, Hospital Complex of Navarra; La Rioja, San Pedro Hospital; M. Valdecilla, Marqués Valdecilla University Hospital. (**A**) Red blood cells concentrate; (**B**) Platelets; (**C**) Fresh frozen plasma.

Figure 2 shows the evolution of the three types of BC in the different centers included, taking as a reference a value of 100 defined as a fortnightly average value, for each component and center, during the months of January and February 2020. A similar pattern of RBCs (Figure 2A) in the different centers is observed, according to the periods analyzed, with a maximum decrease in consumption in all centers during Phase 1 (11–24 May). Regarding the consumption of platelets (Figure 2B) or fresh frozen plasma (FFP) (Figure 2C), the variability between centers is much greater, and no type of analogy can be established between the different periods for each center.

**Figure A1:**
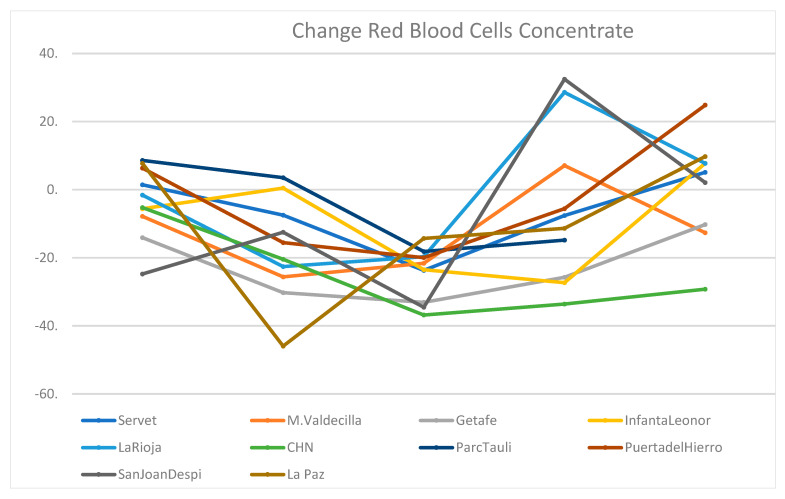
Change in red blood cells concentrate.

**Figure A2:**
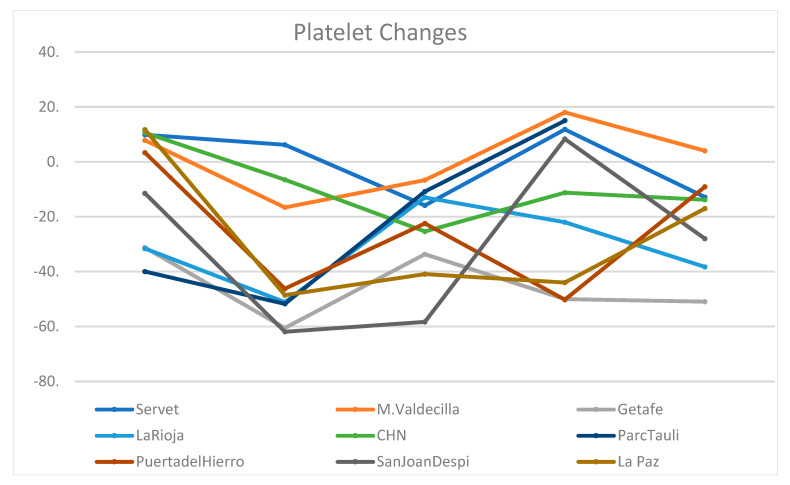
Platelets (excluding Hospital Infanta Leonor due to an “outlier” result of an increase of 310% in Period 0).

**Figure A3:**
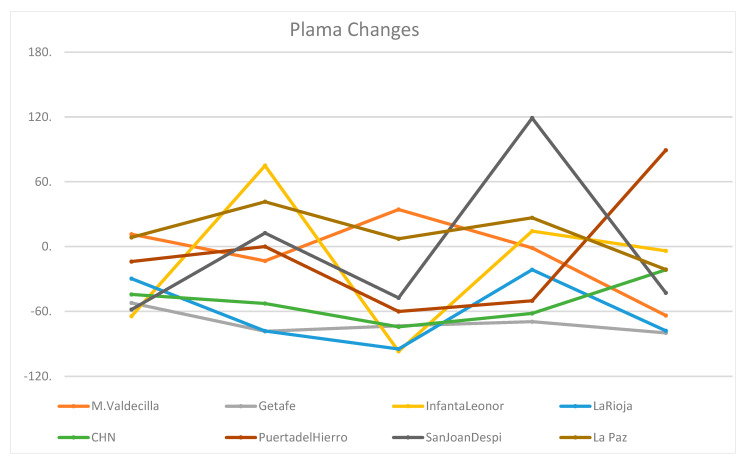
Fresh frozen plasma (excluding the Miguel Servet University Hospital due to an “outlier” for presenting a result of 235% increase in Period 0).

The evolution of blood component transfusion was performed by hospitals, comparing current data with the average of the previous 2 years. No substantial changes were observed in RBCs, with a downward trend in the pre-confinement phase and in Phase 0; in Phases 1 and 2, although variable according to the center, there was an increase (Appendix B, Figure A1A). The consumption of platelets (Appendix B Figure A2) and fresh frozen plasma (Appendix B Figure A3) did not allow us to observe any clear consumption pattern.

### 3.5. Shortage

No hospital reported having suffered a shortage of any product.

### 3.6. Hemovigilance

No CRT received notification of the possible transmission of SARS-CoV-19 by transfusion.

## 4. Discussion

In this study, we have analyzed how the COVID-19 pandemic has influenced blood donations, distribution, and transfusions during different phases of the state of alarm decreed by the pandemic in Spain by comparing the results obtained with the transfusion activity of the previous two years.

At the beginning of the pandemic, there were warnings of possible risk of shortages, and it was recommended to plan activity due to possible supply shortages. To ensure accurate follow-up, the RTCs provided weekly reports on the evolution of donations, stock and the supply, and the exchange capacity. According to these reports (not published to date), donations decreased in Spain by 20% (with a peak of 35% during the Holy Week holidays), with large differences between the autonomous communities. Despite the fact that Spain has been one of the countries in the world with the highest affectation and mortality during the first wave, the observed decrease in donations was much lower than that described internationally in other countries with equal or lesser affectation by COVID-19 [1,2,3,4,5,6,7,8,9,10]. Although the decline was not homogeneous among the autonomous communities analyzed, it was already evident in the fortnight prior to the declaration of the first universal state of alarm and confinement. The decrease was greater in those autonomous communities with higher donation rates [14]; however, a significant upward “rebound” was observed in some autonomous communities in the later phases of confinement, highlighting those with the lowest donation rates [14]. The latter may be logical, if we interpret that the autonomous communities that maintained a better rate of donation may have a lower capacity to recover to the usual levels.

Nevertheless, in Spain, the drop in organ and tissue donations, solid organ transplants, and hematopoietic stem cell transplants was more acute than in other health systems [13]. In addition, in Spain during the first wave of the pandemic and with the endorsement of different scientific societies [12], non-oncological or urgent surgery was suspended for weeks, similar to practices in many other countries [15]. This reduced surgical activity, together with the decrease in transplantation activity during the initial months of the pandemic, could be responsible for mitigating the consequences of the decline in blood donations. In this sense, the first published data on the impact of a pandemic on transfusion practice in southern Italy or Washington revealed a significant decrease in hospital consumption, mainly due to a decrease in surgical demand [9,10], even in day hospitals, although somewhat less [9]. Some centers implemented triage measures for transfusion requests [10,16] to reduce demand and minimize the theoretical risks of transfusion transmission of the virus. This confirms the severe concern of healthcare facilities about a possible shortage, despite the reduction in demand. Additionally, we can see how this concern is shared in different healthcare systems and regions of the world, which have implemented variable measures to adapt to the situation [9,10,15,16].

However, in our study, the transfusion impact of the pandemic has been lower, possibly due to the low transfusion rates of elective surgery, such as orthopedics [17,18]. We believe that this has been the consequence of the need to maintain transfusions for non-delayed interventions or treatments, due to the reorganization of care spaces and personnel, and due to the application of programs for the adequate use of transfusions and alternatives. In a survey conducted on the possible impact of COVID-19 on ERAS (Enhanced recovery after surgery) perioperative care, modifications in preoperative management have been documented in some centers, including a decline in the study and treatment of peri-surgical anemia [19].

The decrease in the supply of BCs in the autonomous communities analyzed was significant as compared with the previous biennium, but only during the confinement phase (16 March–11 May). However, a significant decrease was also observed in the fortnight of March prior to the declaration of the state of alarm. This could be explained because La Rioja and Navarra were among the most affected at the beginning of the pandemic and began to suffer the effects of it before the state of alarm was decreed. This fact could suggest the need to contextualize and adapt the response strategy of the health system to the reality of each environment, since the different speeds of the pandemic between geographical areas can cause substantial differences even between small regions; but this does not mean that the action plan should be different between regions or centers of the same health system.

The consumption of BCs has been highly variable between hospitals, but a drop was observed in all hospitals during confinement. However, in some hospitals, transfusions had already decreased in the previous fortnight, while in others it increased in Phases 1 and/or 2. Although an attempt was made to analyze according to the affectation during the first phase of the pandemic in the surgical activity, admissions, stays, and emergencies attended, we did not find a pattern to explain it. In many of the hospitals, there had been a decrease in previous consumption, but a rebound of more than 20% was observed when leaving confinement. As the drop in donation also limited the availability of blood and BC, surgical interventions had to be delayed and blood components had to be exchanged between autonomous communities, saving measures were taken and “blood management programs” (PBMs) had special relevance [20].

We believe that independent audits or new studies are necessary to interpret what happened with the management of transfused patients during Phases 1 and 2.

The data from the RTCs are well represented and balanced because two RTCs present donation rates, donation rate per donor, plasma collection, and BC consumption above the national average, while the other two RTCs are below the average. In this sense, it is noteworthy that the two RTCs with the highest rates of donation and consumption are precisely those that experienced the greatest drop in the donation and transfusion of RBCs.

Possibly this would reflect a possible habitual overconsumption. According to the data of the ministerial report, it can be inferred that there would be extreme differences among the autonomous communities in the adjusted rates per 1000 inhabitants, (i.e., 50% in the transfusion of RBCs, 100% in the transfusion of platelets, and up to 150% from fresh frozen plasma [14].

Similar differences are observed in donation rates and in obtaining plasma. Such variability in donations and transfusions in a homogeneous health care system could reflect the existence of inappropriate transfusion practices, probably preventable and improvable. This fact has been recognized by the WHO, which, in February 2020, published the “Framework for Action 2020–2023” to achieve “universal access to safe, effective and quality-assured blood products” [21]. In this document, several challenges were identified, such as “(Challenge 5) sub-optimal clinical practices in the transfusion of blood components” or “(Challenge 6) insufficient access to blood during emergency situations”.

This warning from the WHO is in agreement with the results we obtained in our study, i.e., to ensure donations, increased donors, worker and BC safety, and to optimize preventive measures. Along these lines, in some autonomous communities in Spain, the RTCs (in coordination with the Hospital Directorates) issued statements insisting on the optimal use of BCs and recommending PBM measures.

In Spain, the main consumption of blood components occurs in hematology, emergency and intensive care units, then surgical services. Among them are especially cardiac and oncological surgery. These activities were not interrupted, apart from scheduled surgery. There was a reduction in traffic accidents, but not in critical or massive bleeding, due to a greater number of cases of digestive bleeding, and bleeding due to anticoagulation in COVID patients. Faced with the drop in donations, many regions, as seen in countries such as Italy, China, and the United States, had to make calls for donations [22,23].

In Spain, the Spanish Multimodal Rehabilitation Group (GERM), following a national survey, found that the opposite would have occurred during the pandemic [19], with less application of PBM measures [24]. For this reason, GERM has called for the application of evidence-based measures to ensure the improvement of clinical results and a reduction in risks associated with care [21,22,23,25], in line with the WHO proposal for years, i.e., “implementation of the appropriate use of blood products, haemovigilance systems and PBM programs” [21].

This could be one of the main contributions of our study: in times of uncertainty and tension in health centers is when the available evidence should be best and most forcefully applied. With this, we could avoid or reduce excessive practical variability between centers and between regions, in order to try to homogenize the ways of proceeding that affect donations and transfusions. Additionally, for that reason, it is necessary to have a plan that integrates PBM programs could improve variability and the lack of unity in the criteria and decisions to be made [20,21,25].

### Limitations

The number of hospitals selected may be a bias in itself. On the one hand, the inclusion of transfusion data from various hospitals in the autonomous communities such as Catalonia and Madrid (among others), allowed us to reflect a larger transfused population and to mitigate these possible biases. On the other hand, the lack of verified official data on the study parameters forced us to be cautious when generalizing the results obtained. In addition, it is a retrospective study based on activity data from aggregated databases. A possible seasonal effect on donation and transfusion cannot be ruled out.

## 5. Conclusions

The COVID-19 pandemic has affected the donation and consumption of BCs, especially a decrease in RBC transfusions, observed since the fortnight prior to the declaration of the state of alarm. However, a great variability in consumption is observed between autonomous communities and hospitals. With respect to the period prior to the pandemic and the average of the previous biennium, the increases in the consumption of RBCs and platelets during the attenuation phases of the pandemic are noteworthy.

The lack of unified criteria between hospitals and autonomous communities should provoke a reflective process on the part of scientific societies and those responsible for health macro management in order to plan a global strategy on a more transparent hemotherapy and state plasmapheresis plan, with continued benchmarking, and based on the institutional implementation of PBM programs.

## Figures and Tables

**Table 1:** Evolution of donations in the Spanish State *.

	9–15 March	16–22 March	23–29 March	30 March–3 April	6–12 April	13–19 April	20–26 April	27 April–3 May	4–10 May	11–17 May	18–24 May	25–31 May	1–7 June	8–14 June	15–21 June	22–28 June
Week	11	12	13	14	15	16	17	18	19	20	21	22	23	24	25	26
Donation	33,760	30,151	22,595	21,899	20,496	22,395	26,541	24,688	27,480	32,997	32,748	32,174	31,562	32,589	35,239	28,085
Difference	2880	−729	−8285	−8981	−10,384	−8485	−4339	−6192	−3400	2117	1868	1294	682	1709	4359	−2795
(%)	9.3	−2.4	−26.8	−29.1	−33.6	−27.5	−14.1	−20.1	−11.0	+6.9	+6.1	+4.2	+2.2	+5.5	+14.1	−9.1

* Prepared from the weekly reports of the Ministry of Health.

**Table 2:** Comparison of the evolution of donations by communities according to period.

Region		January and February	1–15 March	16 March–10 May	11–24 May	25 May–8 June	11 May–8 June	16 March–8 June
Navarra	2020	3581	800	2294	773	983	1756	4050
	Mean 18–19	3803.5	976	3396.5	867	910.5	1777.5	5174
	Difference (%)	−5.8	−18.0	−32.5	−10.8	8.0	0.1	−21.7
Aragón	2020	7155	1816	5536	1668	1889	3557	9093
	Mean 18–19	7170	1636	5861.5	1723.5	1879.5	3603	9464.5
	Difference (%)	−0.21	11.0	−5.6	−3.2	−0.5	−1.3	−3.9
La Rioja	2020	1663	397	1522	394	470	864	2386
	Mean 18–19	1637	447.5	1598	410	405.5	815.5	2413.5
	Difference (%)	1.6	−11.3	−4.8	−3.9	+15.9	+5.9	−1.1
Cantabria	2020	4139	642	2018	818	793	1611	3629
	Mean 18–19	3752.5	905	3245	810.5	837.5	1648	4893
	Difference (%)	10.3	−29.1	−37.8	+0.9	−5.3	−2.2	−25.8
Total	Average percentage change	1.5 (−9.2, 12.1)	−11.8 (−38.7, 15.1)	−20.1 (−47.9, 7.6)	−4.3 (−12.0, 3.5)	4.8 (−9.9, 19.4)	0.6 (−5.2, 6.4)	−13.1 (−32.9, 6.7)
	*p*-value	0.693	0.256	0.104	0.179	0.377	0.754	0.125

**Table 3:** Evolution of the accumulated consumption of red blood cells, plasma and platelets in hospital centers compared to the same period of the previous biennium.

		1 January–28 February	1–15 March	16 March–10 May	11–24 May	25 May–8 June
Red Blood Cells	Average (%)	−3.6 (−11.0, 3.9)	−17.6 (−28.2, −7.1)	−24.5 (−30.0, −19.1)	−5.8 (−21.9, 10.3)	0.6 (−11.5, 12.7)
	*p*-value	0.307	0.004	<0.001	0.437	0.915
Platelets	Average (%)	2.4 (−25.9, 29.8)	−2.65 (−83.2, 77.9)	−25.3 (−36.5, −14.1)	−16.9 (−37.5, 3.7)	−22.9 (−36.4, −9.4)
	*p*-value	0.846	0.942	0.001	0.097	0.005
Plasma	Average (%)	−15.6 (−53.7, 22.5)	16.0 (−59.4, 91.3)	−45.3 (−76.7, −14.6)	−9.1 (−49.1, 30.8)	−32.9 (−74.1, 8.3)
	*p*-value	0.379	0.638	0.009	0.618	0.103

**Table 4:** Changes in the consumption of concentrated red blood cells by hospital.

		1 January–28 February	1–15 de March	16 March–10 May	11–24 May	25 May–8 June
Navarra	2020	2410.0	554.0	1502.0	717.0	510.0
	Mean 18–19	2543.5	696.5	2405.5	1080.0	720.5
	Difference (%)	−5.25	−20.46	−36.91	−33.61	−29.22
Aragón	2020	2399.00	581.00	1673.00	498.00	597.00
	Mean 18–19	2365.00	628.00	2193.00	539.00	568.00
	Difference (%)	1.44	−7.48	−23.71	−7.61	5.11
La Rioja	2020	1521.00	305.00	1157.00	371.00	397.00
	Mean 18–19	1545.50	394.00	1440.50	288.50	368.50
	Difference (%)	−1.59	−22.59	−19.68	28.60	7.73
Valdecilla	2020	2303.0	463.0	1642.0	597.0	507.0
	Mean 18–19	2499.0	622.5	2095.0	557.5	580.5
	Difference (%)	−7.84	−25.62	−21.62	7.09	−12.66
Parc Tauli	2020	1086	249.00	773.00	224.00	
	Mean 18–19	1000	240.50	944.50	263.00	
	Difference (%)	8.6	3.53	−18.16	−14.83	
San Joan Despi	2020	626	175.00	489.00	202.00	171.0
	Mean 18–19	832.0	200.00	747.00	152.50	167.5
	Difference (%)	−24.76	−12.50	−34.54	32.46	2.09
La Paz	2020	3604.0	714.00	2634.00	667.00	794.0
	Mean 18–19	3346.0	1321.00	3074.50	752.50	723.5
	Difference (%)	7.71	−45.95	−14.33	−11.36	9.74
Getafe	2020	878.0	197.0	620.0	192.0	193.0
	Mean 18–19	1022.0	282.5	926.5	258.5	215.0
	Difference (%)	−14.09	−30.27	−33.08	−25.73	−10.23
Puerta del Hierro	2020	2327.0	483.0	1539.0	499.0	598.0
	Mean 18–19	2188.5	572.0	1923.0	528.5	479.0
	Difference (%)	6.33	−15.56	−19.97	−5.58	24.84
Infanta Leonor	2020	881.0	209.0	547.0	145.0	201.0
	Mean 18–19	933.5	208.0	715.0	199.5	186.5
	Difference (%)	−5.62	0.48	−23.50	−27.32	7.77
Total	Average percentage change	−3.6 (−11.0, 3.9)	−17.6 (−28.2, −7.1)	−24.5 (−30.0, −19.1)	−5.8 (−21.9, 10.3)	0.6 (−11.5, 12.7)
	*p*-value	0.307	0.004	<0.001	0.437	0.915

## Data Availability

The data are available to contact with corresponding author.

## Appendix A

**Table A1:** Distribution of packed red blood cells from the transfusion centers.

		1 January–28 February	1–15 March	16–10 May	11–24 May	25 May–8 June	11 May–8 June	16 March–8 June
Navarra	2020	3573.0.	803.0	2372.0	744.0	719.0	3835.0	1463.0
	Mean 18–19	3740.0	931	3393	851	938	5182	1789
	Difference (%)	−4.5	−13.8	−30.1	−12.6	−23.6	−26.0	−18.2
La Rioja	2020	1521.0	305	1157	371	397	1925	768
	Mean 18–19	1545.5	394	1440	288.5	368.5	2097	657
	Difference (%)	−1.6	−22.6	−19.7	28.6	7.7	−8.2	+16.9
Cantabria	2020	3445.0	718	2259	794	638	3691	1432
	Mean 18–19	3425.0	892.5	2948	775	793	4516	1568
	Difference (%)	0.6	−19.5	−23.4	2.4	−19.6	−18.3	−8.7
Total	Average percentage change	−1.8 (−8.1, 4.7)	−18.6 (−29.7, −7.5)	−24.4 (−37.5, −11.3)	6.2 (−45.6, 57.9)	−11.7 (−53.8, 30.4)	−17.5 (−39.7, 4.7)	−3.3 (−48.4, 41.8)
	*p*-value	0.339	0.019	0.015	0.660	0.354	0.077	0.781

**Table A2:** Platelet distribution from transfusion centers.

		1 January–28 February	1–15 March	16–10 May	11–24 May	25 May–8 June	11 May–8 June	16 March–8 June
Navarra	2020	809.0	186	560	195	182	377	937
	Mean 18–19	630.0	126	568.5	156	170	326	894.5
	Difference (%)	28.4	47.6	−1.5	+25.0	+7.1	+15.6	+4.7
La Rioja	2020	92.0	22	124	23	25	48	172
	Mean 18–19	134.5	45	142.5	29.5	40.5	70	212.5
	Difference (%)	−31.6	−51.1	−13.0	−22.0	−38.3	−31.4	−19.1
Cantabria	2020	568.0	117	443	118	129	247	690
	Mean 18–19	564.5	132.5	503	121.5	131.5	253	756
	Difference (%)	0.6	−11.7	−11.9	−2.9	−1.9	−2.4	−8.7
Total	Average percentage change	−0.9 (−75.5, 73.8)	−5.1 (−128−5, 118.4)	−8.8 (−24.6, 7.0)	0.03 (−58.7, 58.8)	−11.0 (−70.7, 48.6)	−6.1 (−65.0, 52.8)	−7.7 (−37.3, 21.9)
	*p*-value	0.965	0.876	0.138	0.999	0.509	0.701	0.378

**Table A3:** Fresh frozen plasma distribution from the transfusion centers.

		1 January–28 February	1–15 March	16–10 May	11–24 May	25 May–8 June	11 May–8 June	16 March–8 June
Navarra	2020	364	54	179	40	83	123	302
	Mean 18–19	577	114	410.5	112	94	206	616.5
	Difference (%)	−37.0	−52.6	−56.4	−64.3	−11.7	−40.3	−51.0
La Rioja	2020	78	11	16	22	20	42	58
	Mean 18–19	111	50.5	309	28	91	119	428
	Difference (%)	−29.7	−78.2	−94.8	−21.4	−78.0	−64.7	−86.5
Cantabria	2020	285	48	322	39	30	69	413
	Mean 18–19	331	458.5	249.5	70	81.5	151.5	401
	Difference (%)	−13.9	−1.0	29.1	−44.3	−63.2	−54.5	+3.0
Total	Average percentage change	−27.9 (−56.2, 2.4)	−44.0 (−141.6, 53.7)	−40.7 (−198.2, 116.8)	−43.3 (−96.6, 9.9)	−51.0 (−137.4, 35.5)	−53.3 (−83.8, −22.7)	−44.8 (−156.8, 67.1)
	*p*-value	0.059	0.192	0.382	0.073	0.127	0.017	0.227

## Appendix B

Supplementary Figure A1: Evolution of blood component transfusion in hospital centers as compared with the previous biennium. (CHN, Hospital Complex of Navarra; La Rioja, San Pedro Hospital; M. Valdecilla, Marqués Valdecilla University Hospital).
